# Owner and Cat-Related Risk Factors for Feline Overweight or Obesity

**DOI:** 10.3389/fvets.2019.00266

**Published:** 2019-08-19

**Authors:** Meredith Wall, Nick John Cave, Emilie Vallee

**Affiliations:** School of Veterinary Science, Massey University, Palmerston North, New Zealand

**Keywords:** attachment, cat, diet, feline nutrition, feline obesity, owner, personality, psychology

## Abstract

Feline obesity is a highly prevalent disease that poses an urgent and serious challenge. Attempted treatment by weight reduction is often unsuccessful; a new preventative approach that focuses on the role of the owner may be helpful. This study used data collected from an international survey of cat owners designed to assess owner personality and self-control, owner-pet attachment, feeding practices, and the cat's body condition. Owner-reported body condition scores (BCS) of cats were assessed using images adapted from a 5-point BCS system and categorized as a binary dependent variable: overweight/obese (BCS 4–5) and not overweight (BCS 1–3). Owner-reported BCS scores using a verbal BCS scale were also used as a binary dependent variable. Of the 6,835 respondents, 30.5% described their cat as overweight/obese using the visual BCS scale, and 32.5% using the verbal scale. Multivariable logistic regression models were built using stepwise-backward selection. A total of 8 variables were significant using the visual score as the dependent variable, while 11 variables were significant using the verbal score as the dependent variable (*p* < 0.05). Low owner conscientiousness was associated with an increased risk of feline overweight/obesity (OR = 1.23, 95% CI 1.10–1.38), whereas preference for delayed reward was associated with a decreased risk (OR = 0.84, 95% CI 0.75–0.96). Contrary to expectation, indulgent (OR = 0.76, 95% CI 0.53–0.91) and inconsistent (OR = 0.86, 95% CI 0.76–0.93) feeding practices appeared protective. Other significant variables (*p* < 0.05) included cat-related factors (age, gender, housing, source) and management-related factors (dry diet, supermarket dry diet, raw diet, stealing, hunting, and measuring food with a scoop). A third multivariable analysis was performed, using results from cats classified as overweight/obese using both scoring methods, compared with cats classified as a healthy weight using both scoring methods. A total of 10 variables were found to be significant (*p* < 0.05). There was significant overlap of results from all three analyses. The results of this study indicate that feline obesity is a complex problem, with many contributing risk factors. It is essential to recognize the importance of owner characteristics, and that the prevention of obesity in cats may require the development of a range of interventional strategies.

## Introduction

Obesity is currently one of the greatest health and welfare problems facing domestic cats around the world. Multiple, recently published studies suggest that in developed countries, anywhere from 11.5 to 63% of pet cats are overweight or obese (1–7). There are many reasons why an increased focus on obesity prevention in cats is critical. Firstly, attempted weight reduction in later life often fails, with many cats either failing to lose weight, or to maintain a healthy weight (8, 9). Secondly, many established risk factors for obesity (such as neutering or indoor confinement) actually provide substantial benefit to the owner and the animal itself (10, 11). Finally, the consequences and costs of obesity are well-established in many species, and include the development of multiple associated diseases, reduced quality of life and a decrease in lifespan (12, 13). A new approach to obesity prevention in cats is clearly needed.

This approach should have an increased focus on the owner and their behavior, because the owner is responsible for the great majority of the cat's food intake. To date, relatively little is known about the factors that may cause owners to overfeed their cats, however there is extensive research demonstrating that certain parental characteristics can increase the likelihood of excessive weight gain in their children. Psychological dispositions, indicated by measures of personality and behavior, have been strongly implicated in obesity risk in both adults and their dependent children (14, 15). Of particular interest is the association between self-control, personality traits, impulsiveness, and obesity.

A high level of self-control, or the ability to resist short-term temptations in order to achieve long-term objectives, has been shown to be associated with health-oriented behaviors in people, such as increased physical activity and consumption of a healthy diet (16, 17). This may partially be because good self-control positively predicts increased eating consistency, which refers to adherence to a similar diet in different circumstances (18). Adults that consume a similar number of calories each day have be shown to have lower body fat mass, and lower fat and energy intake, compared to those with more inconsistent caloric intake (19). An inconsistent diet and poor self-control are also associated with greater impulsivity and a strong preference for immediate reward, both of which are established risk factors for excessive weight gain and obesity in people (20–22).

Restrained eating and healthy body weight are also correlated with particular personality traits, such as higher conscientiousness, extraversion and openness, and lower neuroticism (23). More significantly, parental personality appears to shape the feeding practices that parents use, with mothers of obese children scoring lower on conscientiousness (15). It therefore appears that there is an association between high conscientiousness, high self-control, low impulsivity and healthy-weight in adults. Given that these psychological characteristics in parents have also been shown to affect the obesity risk of their dependent children, it is hypothesized that comparable associations may be found in cat owners and their cats.

As far as the authors are aware, the complex relationship between owner psychology and the risk of feline obesity has not yet been comprehensively explored. The aim of this study was, therefore, to determine if there are particular owner psychological factors that are strongly correlated with the development of overweight or obesity in cats. It was hypothesized that a low level of owner conscientiousness, low owner self-control, owner preference for immediate reward, high owner attachment (to the cat) and the use of indulgent or inconsistent feeding practices would be associated with an increased risk of feline obesity. Identifying these risk factors may assist in developing new approaches to obesity prevention in cats that focus on understanding owner psychology, in order to successfully modify attitudes and behaviors.

## Materials and Methods

### Subjects

Data for this cross-sectional study were obtained from a multinational, online questionnaire designed to assess owner psychology and socio-demographics, cat health, and feeding practices (Supplementary Material A). Inclusion criteria were that respondents must be aged 18 years or over and own at least one pet cat. For multi-cat households and breeders, the owner was asked to provide information about one cat only, and was asked to select the cat whose name begins with the letter closest to the start of the alphabet, in an attempt to avoid selection bias.

To determine the number of participants required, cross-sectional studies on the same, or similar, risk factors for obesity in adults and children were reviewed, which suggested a minimum number of ~5,500 respondents (24). These risk factors included personality traits, self-control, indulgent feeding practices and preference for immediate reward. This estimate corresponded well with a calculated sample size of 5,496, using a confidence level of 99%, margin of error of 1.74 and estimated population of 35 million cat owners in the United States (25). It is well-recognized that feline obesity, like obesity in humans, is a complex disease with many contributing factors, so recruiting a large number of participants was important to allow detection of relatively small effect sizes. Furthermore, as discussed in section Dependent variables, there is likely to be inherent bias in studies that use owner-reported body condition score (BCS) as a dependent variable; a larger sample size improves the potential to detect significant associations when misclassification may be occurring.

### Data Collection

The online questionnaire (Supplementary Material A) contained 27 questions or measures in eight parts, which were designed to assess owner personality, self-control and attachment to their cat, feeding practices, cat health and body condition, and owner socio-demographics.

Part One assessed the cat's health and body condition (further described in section Dependent variables), as reported by the owner. Part Two of the questionnaire evaluated the owner's personality traits, using the Big Five Inventory with 10 items (BFI-10). This is an abbreviated version of the Big Five Inventory with 44 items (BFI-44), developed for use in research settings when time for questionnaire completion is limited (26). The questionnaire consists of 10 items divided into the five broad domains—neuroticism, extraversion, openness, agreeableness, and conscientiousness, each of which are rated on a 5-point Likert scale.

Part Three contained the Lexington Attachment to Pets Scale (LAPS). The Lexington Attachment to Pets Scale, developed by Johnson, Garrity, and Stallones, is perhaps the most widely used questionnaire to assess emotional attachment to pets (27). Part Four of the questionnaire contained the 13-item brief Self-control Scale (SCS), as developed by Tangney et al. (28). It was designed to assess people's ability to override or alter internal responses, and to interrupt undesired behavioral inclinations and refrain from acting on them.

Part Five of the questionnaire was the Consideration of Future Consequences (CFC-14) scale, which was originally designed to assess the extent to which people emphasize short-term or long-term consequences. Petrocelli (29) and Joireman et al. (30) determined that the CFC scale contains two underlying sub-scales: concern with immediate vs. concern with future consequences (29, 30). The authors' exploratory and confirmatory factor analyses of the CFC-14 scale supported the presence of two highly reliable factors (CFC-Future and CFC-Immediate). In our study, the two sub-scales were therefore scored and analyzed separately.

Part Six assessed the extent to which the owner employs an indulgent feeding style when feeding their cat. There are several psychometrically robust questionnaires that have been used to evaluate the types of parental feeding styles and practices that increase the risk of weight gain in young, dependent children. Examples of these include the Feeding Practices and Structure Questionnaire (31), the Toddler Feeding Behavior Questionnaire (32) and the Caregiver Feeding Style Questionnaire (33). Unfortunately, no such questionnaire has been developed for veterinary use.

Therefore, the degree of indulgence with respect to feeding was assessed using the Indulgent Feeding Style Questionnaire, specifically developed for this study. The items for the questionnaire were derived from a review of the clinical and experimental literature on parental feeding behaviors, adapting existing human questionnaires (in particular, the Toddler Feeding Behavior Questionnaire) and carrying out informal interviews with a small convenience sample of cat owners (31, 32).

Part Seven assessed the owners' daily feeding practices, such as what they currently feed their cat and how consistent their feeding routine is. This section predominantly contained straightforward multiple choice questions that were quick and easy for the owner to answer. Finally, Part Eight briefly assessed key socio-demographic characteristics of owners.

The online questionnaire was written in English and designed to take participants around 20 min to complete. The questions were all “closed” questions with multiple-choice answers. The questionnaire was piloted on a small number (*n* = 20) of cat owners to ensure that the time taken for completion, question order and wording, and level of language were all appropriate for the target audience. Given that the psychological scales used were already widely accepted, the validity and reliability of the questionnaire was not further assessed.

Respondents were recruited between March 2017 and May 2017; recruitment occurred mainly via social media. The questionnaire was posted in different social media groups relating to pet ownership, and a range of hobby groups (for example, sports groups, craft groups, gardening and cooking groups, and music-related groups). The authors attempted to minimize non-response bias by offering a small incentive to complete the questionnaire (one prize to the value of $100 NZD, consisting of feline care products). All responses were anonymous, with all personal respondent information (first name, last name, email address, and IP address) excluded from results.

### Dependent Variables

The primary dependent variable used for the study was owner-reported body condition score (BCS). This was the only practical method of detecting feline overweight or obesity as part of a large, international questionnaire. This study used the same methods to assess owner-perceived BCS as Colliard et al. (4), whereby owners were asked to evaluate their cat's BCS using two methods—by verbal description and by a visual scale (4). The visual scale was adapted from the World Small Animal Veterinary Association (WSAVA) Body Condition Score chart^1^ for cats, and consisted of five legend-free drawings of cats of increasing BCS, randomly arranged in a circle (34). A verbal BCS question followed on the next page of the questionnaire, after owners completed the visual scale question. The primary reason for this was to assess the difference between results using the visual scale, and results using the verbal scale.

In order to minimize any misclassification (as discussed in section Dependent variable), this study converted owner-reported BCS to a binary dependent variable: overweight or obese (BCS 4–5) and not overweight (BCS 1–3). This categorization was performed for both the visual scale question and verbal description question. For clarity and simplicity, the term “obese,” as used in this study, will refer to cats with a BCS of either 4 or 5, rather than the term “overweight or obese.”

### Explanatory Variables

Eighty-four explanatory variables were extracted from the data and analyzed as potential risk factors for feline obesity. These variables are summarized in Supplementary Material B.

### Statistical Analysis

The primary purpose of the analyses was to determine which explanatory variables were associated with obesity in cats. Eight thousand four hundred one respondents either completed or partially completed the questionnaire. One thousand five hundred sixty-seven respondents failed to complete every question; their responses were therefore excluded from all analyses. This resulted in 6,835 complete responses remaining.

Numerical scores of the BFI-10 scale were calculated and different personality traits were analyzed as categorical variables. Similarly, numerical scores from the LAPS, SCS, CFC-14 scale and IFSQ were calculated and converted to categorical variables for logistic regression.

Initially, univariable logistic regression models were used to screen explanatory variables for an association with the outcome of feline obesity (obese = 0 or 1). Multicollinearity was assessed by computing the variance inflation factor (VIF) values for all variables, as described in Dohoo (35). One diet-related variable (being fed a weight loss diet) was removed from the model, as it returned a VIF value >10, suggesting possible multicollinearity. VIF values for all other variables were <10; these variables were therefore included in the models. Multivariable logistic regression was then performed using those variables shown to be significant in the univariable analysis (*p* < 0.20). Stepwise backward selection was used to identify significant explanatory variables; the alpha level for determination of significance was 0.05. A likelihood ratio test was used to compare nested models. Assumptions of statistical models were checked graphically, also as described in Dohoo (35). Finally, biologically plausible interactions were tested for significance, and the goodness-of-fit of the models was evaluated with the le Cessie-van Houwelingen-Copas test (36). All analyses were carried out with the R version 3.3.1 statistical programming language (37).

## Results

### Dependent Variables

There was a total of 6,835 responses to the visual BCS assessment question, following the exclusion of incomplete or partially completed questionnaires. Of these, 30.5% (*n* = 2,087) described their cat as overweight or obese. Similarly, 32.5% (*n* = 2,221) described their cat as overweight or obese when answering the verbal BCS assessment question. It was found that 16.0% of cats (*n* = 1,094) had discordant results with respect to the visual and verbal scales (Table 1). Of those, 23.0% of cats (480/1,094) scored as overweight or obese using the visual scale, were scored as not overweight or obese using the verbal scale. Similarly, 27.6% of cats (614/1,094) scored as overweight or obese using the verbal scale, were scored as not overweight or obese using the visual scale. Finally, 23.5% of all respondents (*n* = 1,607) scored their cat as overweight or obese using both the visual and verbal scales, whereas 39.5% of all respondents (*n* = 2,701) scored their cat as overweight or obese, on either one or both scales.

**Table 1:** Comparison of owner-reported body condition score of cats using two methods – the visual scale and the verbal scale.

	**BCS (verbal scale)**	
	Not overweight	Overweight
**BCS (visual scale)**		
Not overweight	4,134	614
Overweight	480	1,607

*Results were categorized as a binary dependent variable: overweight or obese (BCS 4–5) and not overweight (BCS 1–3). This categorization was performed for both the visual scale question and verbal description question*.

### Descriptive Statistics

Respondents from 81 countries completed the questionnaire, with the majority of respondents coming from the United States, Australia, the United Kingdom and New Zealand. The great majority of respondents were female (91.7%, *n* = 6,267) and 7.3% (*n* = 502) were male, with a small number of owners (*n* = 66) preferring not to provide their gender. Most owners lived in cities or towns, with only 9.9% (*n* = 674) living in a rural environment.

Personality traits of cat owners followed a normal distribution, with mean scores and standard deviation for each trait very similar to the general population (38). Mean scores, standard deviation and distribution for self-control, preference for immediate reward and preference for delayed reward were also similar to previously reported studies assessing these variables in adult undergraduate students in the United States (28, 39).

Ownership of male and female cats was very similar, with 49% female cats and 51% male cats. The great majority of cats were neutered (93.9%, *n* = 6,422). With respect to housing, nearly half of all respondents indicated that their cats lived indoors only (48.9%, *n* = 3,347). A large number of cats in this study were acquired from a registered breeder (17.1%, *n* = 1,168); this was second only to cats acquired from rescue groups or centers (28.2%, *n* = 1,928).

Dry food was the most popular type of diet fed to cats, with 91% of owners (*n* = 6,220) feeding dry food as the entire diet, or as a component of the diet. Of those owners, 31.6% (1,967/6,220) fed dry food purchased from a supermarket, 36.5% (2,272/6,220) fed dry food purchased from a pet store and 17.1% (1,066/6,220) fed dry food purchased from a veterinary clinic. It was found that 20.6% of owners (1,278/6,220) elected to feed grain-free dry food. The most common reasons to feed a dry food diet were: perceived health benefits (42.8%); convenience and ease of feeding (40.8%); vet recommendation (24.5%) and owner perception that the cat preferred crunchy foods (17.6%).

The results indicated that 69.3% of owners (n = 4,739) fed canned foods either intermittently or as the entire diet. Of these owners, 33.9% (1,608/4,739) fed canned food purchased from a supermarket, making it the second most popular type of diet overall. Only 10.4% of all owners (*n* = 711) fed a range of canned foods as the entire diet. The most common reasons for owners to select a canned diet were: perceived health benefits (31.6%); it was the cat's favorite diet (27.2%); owner perception that the cat preferred soft foods (20.0%); and owner perception that the food appeared tasty (15.8%).

Mixed feeding practices (offering both dry and canned foods) were relatively common, with 33.9% of owners (*n* = 2,316) indicating that they consistently fed the same dry food, with a range of canned foods. A large number of respondents (46.3%, n = 3,166) indicated that they fed their cat exactly the same food every day. With respect to raw meat-based diets, 9.7% of all respondents (*n* = 666) fed a commercial raw diet, while 15.5% (*n* = 1,062) fed human-grade meat to their cat, either as a component or as the entire diet. Of the 25.3% of owners (*n* = 1,728) feeding some type of raw diet, slightly less than half of these (40.4%, *n* = 699) indicated that they exclusively fed a raw diet, which equates to 10.2% of all respondents (699/6,835).

The results revealed that 36.3% of people (*n* = 2,478) believed that they were not able to control what their cat eats on a daily basis. The main reasons for this were hunting and eating prey (51.7%), stealing human food (26.6%) and stealing another cat's food (26.2%). Finally, owners employed many different methods with respect to deciding how much to feed their cat. The most popular methods were: always making food available (37.5%, *n* = 2,563); using a measuring cup (27.0%, *n* = 1,845); following package recommendations (24.2%, *n* = 1,656); and following a veterinarian's recommendations (23.4%, *n* = 1,597).

### Univariable Analysis

There were 50 variables significantly associated with feline obesity, as assessed by the visual scoring method and using a critical value of *p* < 0.20. Using the verbal scoring method, obesity was associated with 56 variables. These variables are available as Supplementary Materials C, D and were taken forward to the multivariable analyses. A third univariable logistic regression model used only results from cats scored as obese using both the visual and verbal scoring methods as the dependent variable; this was compared with only cats scored as not obese using both methods (Supplementary Material E). Fifty-four variables were significantly associated with feline obesity and were taken forward to the third multivariable analysis.

### Multivariable Analysis

Two multivariable analyses were performed, using either the visual (Model 1, Table 2) or the verbal (Model 2, Table 3) BCS as the dependent variable. There were 8 significant variables (*p* < 0.05) using the visual score as the dependent variable, while 11 variables were significant using the verbal score as the dependent variable.

**Table 2:** Model One: multivariable logistic regression model results for risk factors for feline obesity (using results for owner-reported visual BCS as the dependent variable).

**Variable name**	**Category**	**Odds ratio (95% CI)**	***P*-value for variable (LRT)**
Age	<1 years 1–4 years 5–8 years 9–12 years 13–16 years > 16 years	Ref. 2.73 (2.10–3.61) 5.01 (3.82–6.66) 5.05 (3.81–6.79) 4.02 (2.96–5.53) 2.16 (1.44–3.22)	<0.001
Gender	ME FE MN FN	Ref. 0.77 (0.43–0.94) 1.40 (1.13–2.26) 1.19 (1.02–1.96)	0.002
Source	Registered breeder Unregistered breeder Pet store Friend/family Rescue group/shelter Stray Pound Private online seller	Ref. 1.47 (0.97–2.22) 1.68 (1.17–2.38) 2.05 (1.59–2.45) 1.86 (1.50–2.18) 1.88 (1.52–2.33) 2.04 (1.51–2.58) 1.94 (1.36–2.44)	<0.001
Owner conscientiousness	High Low	Ref.1.13 (1.01–1.27)	0.044
Owner preference for delayed reward	Low High	Ref.0.84 (0.75–0.96)	0.006
Diet	Dry food—supermarket No dry food fed Raw meat (human grade only) Freeze-dried food Home-prepared	1.23 (1.09–1.39) 0.64 (0.50–0.81) 0.65 (0.54–0.77) 0.66 (0.47–0.91) 0.65 (0.47–0.89)	<0.001 <0.001 <0.001 0.012 0.008
Control over feeding	Cat hunts Cat steals human foods Cat steals other cat's food	0.71 (0.62–0.83) 0.67 (0.54–0.82) 1.55 (1.29–1.87)	<0.001 <0.001 <0.001
Feeding method	Measure food with scoop Weigh food on scales Adjust amount for cat's body weight	1.15 (1.01–1.30) 0.74 (0.56–0.98) 0.79 (0.68–0.91)	0.032 0.028 <0.001

**Table 3:** Model Two: Multivariable logistic regression model results for risk factors for feline obesity (using results for owner-reported verbal BCS as the dependent variable).

**Variable name**	**Category**	**Odds Ratio (95% CI)**	***P*-value for variable (LRT)**
Age	<1 years 1–4 years 5–8 years 9–12 years 13–16 years >16 years	Ref. 3.42 (2.59–4.59) 6.40 (4.82–8.65) 5.94 (4.41–8.12) 4.34 (3.15–6.07) 2.61 (1.73–3.93)	<0.001
Gender	ME FE MN FN	Ref. 0.57 (0.29–0.98) 1.78 (1.08–3.10) 1.67 (1.01–2.91)	<0.001
Housing	Indoors/outdoors Indoors only Indoors/restricted outdoors Outdoors/restricted indoors Outdoors only	Ref. 1.18 (1.03–1.35) 1.37 (1.17–1.61) 0.49 (0.22–0.98) 0.50 (0.11–0.91)	<0.001
Source	Registered breeder Unregistered breeder Pet store Friend/family Rescue group/shelter Stray Pound Private online seller	Ref. 2.06 (1.36–3.07) 2.16 (1.52–3.08) 2.02 (1.62–2.52) 2.14 (1.77–2.60) 2.31 (1.86–2.87) 2.58 (1.97–3.37) 2.19 (1.63–2.93)	<0.001
Owner conscientiousness	High Low	Ref.1.23 (1.10–1.38)	<0.001
Indulgent feeding	Low High	Ref.0.74 (0.53–0.88)	<0.001
Consistent feeding	High Low	Ref.0.86 (0.76–0.93)	0.036
Diet	No dry food fed Same food everyday Raw meat (human grade) Freeze-dried food Home-prepared Supplements	0.67 (0.54–0.83) 1.14 (1.01–1.28) 0.79 (0.67–0.93) 0.72 (0.52–0.99) 0.58 (0.41–0.81) 0.77 (0.60–0.96)	<0.001 0.030 0.007 0.046 <0.001 0.024
Canned diet	Appealing to owner Perceived health benefits Breeder's recommendation	1.19 (1.02–1.38) 0.79 (0.71–0.89) 0.63 (0.39–0.99)	0.026 <0.001 0.045
Control over feeding	Cat hunts/eats prey Cat steals other cat's food	0.78 (0.67–0.90) 1.46 (1.22–1.75)	<0.001 <0.001
Feeding method	Measure with scoop	1.24 (1.10–1.41)	<0.001

Model 3 (Table 4) reports risk factors identified for cats classified as obese using both scoring methods, compared with cats classified as a healthy weight using both scoring methods. This removed any cats with discordant results from the analysis. Ten variables were found to be significant (*p* < 0.05). The variables that were found to be significant in all three models were: the cat's age, cat's gender, cat's source, owner conscientiousness, not feeding dry food, feeding raw meat (human grade), stealing another cat's food, and measuring food with a scoop. There were no significant interaction terms for any of the biologically plausible interactions (24 were tested) in any analysis. All three models returned *p*-values for the le Cessie-van Houwelingen-Copas test of 0.3–0.7 (Model 1 *p* = 0.62, Model 2 *p* = 0.47, Model 3, *p* = 0.58), showing no significant difference between predicted and observed probabilities, hence indicating an acceptable goodness of fit of the three models (36).

**Table 4:** Model Three: Multivariable logistic regression model results for risk factors for feline overweight or obesity (using results from all cats scored as overweight using both the visual and verbal scoring methods [*n* = 1,607], compared with all cats scored as not overweight using both methods [*n* = 4,134]).

**Variable name**	**Category**	**Odds Ratio (95% CI)**	***P*-value for variable (LRT)**
Age	<1 year 1–4 years 5–8 years 9–12 years 13–16 years >16 years	Ref. 5.10 (3.37–8.07) 11.76 (7.74–18.68) 11.10 (7.21–17.84) 7.12 (4.51–11.67) 4.15 (2.34–7.48)	<0.001
Gender	ME FE MN FN	Ref. 0.64 (0.25–0.96) 1.77 (1.08–3.78) 1.64 (1.12–3.52)	0.002
Housing	Indoors/outdoors Indoors only Indoors/restricted outdoors Outdoors/restricted indoors Outdoors only	Ref. 1.23 (1.03–1.48) 1.45 (1.18–1.80) 0.20 (0.14–0.57) 0.54 (0.11–0.95)	<0.001
Source	Registered breeder Unregistered breeder Pet store Friend/family Rescue group/shelter Stray Pound Private online seller	Ref. 2.28 (1.29–3.94) 2.91 (1.85–4.56) 2.71 (2.04–3.61) 2.45 (1.92–3.17) 2.71 (2.05–3.59) 2.49 (1.75–3.55) 2.18 (1.48–3.21)	<0.001
Owner conscientiousness	High Low	Ref.1.23 (1.07–1.41)	0.004
Indulgent feeding	High Low	Ref.1.27 (1.10–1.47)	0.001
Diet	Supermarket dry food No dry food fed Same food everyday Raw meat (human grade) Raw bones Home-prepared	1.22 (1.04–1.42) 0.56 (0.41–0.76) 1.23 (1.07–1.42) 0.69 (0.54–0.87) 0.58 (0.36–0.92) 0.49 (0.29–0.79)	0.007 <0.001 0.005 0.003 0.022 0.003
Canned diet	Breeder's recommendation	0.51 (0.23–0.99)	0.047
Control over feeding	Cat steals from neighbors Cat steals human foods Cat steals other cat's food	1.58 (1.11–2.25) 0.71 (0.55–0.92) 1.63 (1.28–2.07)	0.012 0.019 <0.001
Feeding method	Measure food with scoop	1.21 (1.03–1.42)	0.019

## Discussion

### Dependent Variable

This study employed two methods to assess owner-reported BCS—a visual scale and a verbal scale. The number of cats reported as overweight or obese using both methods (30.5 and 32.5%, respectively), indicates a similar prevalence to other recent studies in Australia, the United States and Great Britain (7). For example, a recent study by Rowe et al. (40) recorded both owner-reported BCS and vet-reported BCS for a small subsample (40). According to vet-reported BCS, 36.8% of cats were overweight or obese (53/144), while 37/144 (25.7%) cats were classified as overweight or obese by their owners.

There are multiple reasons why underrepresentation of overweight cats may have occurred in this study population. Firstly, cat owners who elect to participate in an online questionnaire may be more observant and conscientious with respect to their cat's health and weight, compared with the general population. Secondly, some cat owners with an overweight cat may be aware of the problem but too embarrassed to report it, and therefore be more likely to score their cat as having normal body condition. Thirdly, previous research suggests that many owners are fundamentally inaccurate with respect to how they perceive their cat's body condition. BCS is frequently underestimated, and these owners appear to be unaware that their cat is overweight (3–5, 41).

This may be evidence of “visual normalization”; overweight cats are increasingly common in many countries, and are also often promoted as “cute,” “cuddly,” or “funny” by the media (13). It is possible this might have led to a change in owner perception of what “normal” feline body condition is. This phenomenon has been reported in human studies, with large numbers of parents of overweight or obese children failing to perceive their child as being overweight (42, 43). For these reasons, therefore, some misclassification of BCS by owners was anticipated based on previous research, with overweight and underweight cats more likely to be misclassified than cats with an ideal body condition. This misclassification is a recognizable source of information bias; there is a risk this may conceal associations of interest. It is acknowledged that these biases are a limitation of this study, however, the likely outcome is that the magnitude of any associations detected may be underestimated (44).

It is possible that there may be a lesser degree of misclassification when owner-reported body condition scores are converted to a binary dependent variable—overweight/ obese vs. all other cats. Double sampling is a method that combines a small validation sample with an error-prone main-study sample, potentially yielding results that are more accurate (45). Rowe et al. (40) employed this method in their study on early-life risk factors for feline obesity; 144 cats (of 375, 38%) had both an owner-reported BCS and a vet-reported BCS. The authors' found that there was significant agreement between the owners and vets (*n* = 144, kappa = 0.299, *p* < 0.0005) and concluded that owner-reported BCS (as a binary dependent variable) can be considered a fair representation of vet-reported BCS (40).

### Owner-Related Risk Factors for Feline Obesity

Of the owner-related risk factors assessed by the questionnaire, personality (level of conscientiousness), preference for immediate vs. delayed reward, indulgent feeding practices, and consistent feeding practices were found to be significant.

Existing research suggests that, in people, personality traits are closely linked to body weight, body mass index (BMI) and obesity risk (46, 47). Many past studies assessing Big Five personality traits have suggested that, in people, conscientiousness is protective against obesity (48). Additionally, Sutin and Terracciano found that children with obesity had mothers who scored lower in conscientiousness (15). The results of our study confirmed that cat owners with low scores for conscientiousness are at increased risk of owning an overweight or obese cat (Model 3: OR 1.23, *p* < 0.001). In people, high conscientiousness is associated with self-discipline, diligence and organized meal planning (49). It is possible that cat owners with these traits are more able to appropriately monitor and regulate their cat's food intake, and less likely to feed their cat impulsively in response to begging.

Owner personality traits may have important implications for the success of feline weight management programs. Multiple studies have revealed that there is a clear association between conscientiousness and adherence to medical recommendations (50, 51). This may suggest that particular types of conscientious cat owners will be more responsive to clear and concise scientific information regarding obesity and its detrimental effects, as well as their veterinarian's dietary and weight management recommendations. Perhaps more significantly, it may be quick and simple to identify cat owners with lower overall conscientiousness, using the BFI-10. This could assist with early recognition of cats or kittens that are at an increased risk of becoming overweight or obese, so that appropriate intervention can be made. These interventions could include more intensive monitoring of body weight and BCS, the use of growth curves to try and identify early rapid growth, and recommendation of diets formulated especially for neutered animals (52).

Higher conscientiousness has also been shown to correlate positively with lower impatience, which is significant as it implies that stable personality traits may be strongly associated with another risk for factor for obesity or overweight in people: a preference for immediate, rather than delayed, gratification (53). Using visual BCS as the dependent variable, this study also found owner preference for delayed reward to be associated with a decreased risk of feline obesity (Model 1: OR = 0.84, *p* = 0.005).

A possible explanation for this is that the rewards of preventing feline obesity are, indeed, likely to be delayed. There is often a very large temporal distance between the period when obesity typically develops in cats (early life) and the period of time when owners may see the benefit of earlier restraint (absence of a particular disease, for example). This may mean that veterinarians need to focus less on emphasizing the long-term benefits of weight management and instead highlight the immediate benefits of obesity prevention, such as increased play and activity, performance of natural feline behaviors such as climbing or scratching, ability to groom normally and maintenance of a healthy coat, and slower progression of osteoarthritis and a better quality of life.

Models 2 and 3 revealed that highly indulgent feeding was associated with a decreased risk of obesity. This was unexpected, given that across an extensive series of studies, an indulgent parental feeding style has been linked to higher child body mass index (BMI) scores (54, 55). Parents with an indulgent feeding style commonly use food rewards, or let their child eat whenever or whatever they like. Cat owners that scored highly with respect to indulgent feeding perform similar actions, such as treat feeding, offering multiple different meals, offering special foods on special occasions, and allowing the cat to choose when to eat.

There are a number of reasons why indulgent feeding practices may be associated with decreased risk of obesity in cats. Cat owners that are willing to overlook the expense and inconvenience of quickly replacing an uneaten meal with an alternative, or offering special meals at certain times, may be highly dedicated owners that are fundamentally more attentive to their cat's health and body condition. Alternatively, owners that employ more indulgent practices may be feeding more expensive diets or treats. It is possible that they are then more careful with respect to the amount fed, in order to minimize cost.

Also unanticipated was the result that owners feeding a less consistent diet are at lower risk of owning an obese cat. This was indicated not only by the results of the three consistent feeding questions, but also a separate question confirming that cats fed the same food everyday were at increased risk of obesity (Model 3: OR 1.23, *p* = 0.004). This result was surprising, given our hypothesis that cat owners that are more consistent in their feeding practices may be less at risk of overfeeding their cat unintentionally. Additionally, several studies demonstrate that people consuming a similar number of calories each day have lower body fat percentages, and lower fat and energy intakes, compared to those with more inconsistent caloric intake (19).

Inconsistent feeding practices may be associated with a decreased risk of obesity in cats for several reasons. Feeding a wide variety of different foods may decrease boredom and begging, which may subsequently decrease owner temptation to overfeed their cat. Additionally, unfamiliar foods may be less readily accepted by many neophobic cats, consistently decreasing overall intake (56, 57). Similar to owners that feed a highly indulgent diet, owners that take the time to offer a wide range of foods may be more motivated and attentive, resulting in greater attention to their cat's appearance and body condition.

In 2006, Kienzle and Bergler found evidence of a closer relationship between obese cats and their owners, which contrasted with our finding that the owner's attachment to their cat was not a significant risk factor for obesity (41). This may simply be a result of the larger number of participants in this study, and therefore greater power to detect a true effect. However, it is also possible that in the intervening time period between the two studies, the nature and consequences of strong owner attachment has changed, whereby a greater percentage of highly-attached cat owners are more educated and invested in their animal's health.

### Cat-Related Risk Factors for Feline Obesity

Four cat-related risk factors were found to be significant in the multivariable analyses; these were the cat's age, gender, housing and source. With respect to the cat's age, the results indicated that middle-aged cats (8–12 years) are at highest risk of obesity, when compared with very young cats and very elderly cats. This is not surprising given the results of previous studies, and confirms that a focus on early-life prevention of obesity is essential (5, 58).

Results regarding the cat's gender and neuter status also corresponded with previous studies; in our study neutering was found to be a risk factor for obesity in both male and female cats, using both the visual and verbal scores as dependent variables (3, 59, 60). It is well-established that removal of gonadal estrogen dramatically increases appetite in both males and females for the 6–12 month period following neutering, subsequently increasing the risk of obesity (61, 62). Preventative efforts should be therefore be directed toward owners at time of neutering.

Housing was also significantly associated with obesity or overweight using the verbal score as the dependent variable. Cats that were housed either exclusively outdoors, or outdoors with restricted indoor access, were much less likely to be overweight or obese, when compared with cats housed indoors only, or predominantly indoors. This finding is consistent with previous research and seems logical for three main reasons (44). Firstly, outdoor cats are likely to find their environment more mentally stimulating, which may decrease begging and overeating due to boredom. Secondly, for cats living in multi-cat homes that prone to anxiety, the outdoors may actually be significantly less stressful. It is well-established in other species that chronic social stress can lead to increased calorie intake and adiposity (63, 64). Obesity has been epidemiologically associated with obstructive and non-obstructive feline idiopathic cystitis; chronic stress and anxiety may be important risk factors for both conditions (65, 66). Thirdly, increased activity or exercise due to outdoor housing may also play a small role in moderating appetite and food intake, and increasing energy expenditure (67).

All three models confirmed that cats purchased from a registered breeder were at greatly decreased risk of being overweight, when compared with cats acquired from all other sources. These results are similar to those of Colliard et al. which indicated that purebred cats have a lower risk of obesity, and Domestic Shorthair and crossbred cats are at greater risk of obesity (4). When using the verbal scores as the dependent variable (Model 2), a recommendation by the breeder to feed a canned diet was also significantly associated with a decreased risk of obesity (OR 0.63, *p* = 0.045), highlighting the important role that breeders could play in terms of obesity prevention. Providing new owners with information on diet, different feeding methods, body condition and environmental enrichment are all essential tasks that could be performed by breeders at point of sale. Additionally, many new owners maintain contact with their cat's breeder and turn to them throughout the animal's life for support and advice. The results of this study indicate that dedicated and informed breeders could have a major role to play with respect to obesity prevention, and further research on this area is needed.

It is possible that the association between purchasing a cat from a registered breeder and the risk of overweight or obesity may be confounded by the types of breeds making up this population. It is a limitation of this study that the cat's breed was not recorded as part of the survey, because several studies suggest that there may be a higher incidence of obesity in certain cat breeds (4, 68). Higher BCS have been recorded for British Shorthair and Persian cats, whereas lower BCS have been recorded for Abyssinian, Cornish Rex, Devon Rex, Oriental Shorthair and Sphynx cats (69). It has been hypothesized that this may be because certain breed standards (for example, the Persian breed standard) favor cats with an overweight appearance, by esteeming cats that are “massive,” “heavily boned,” “cobby,” and “well-rounded” (70).

In this study, almost all cats purchased from a registered breeder were located in the United States, Australia, the United Kingdom or New Zealand. In 2017 in the United States, the Exotic, Ragdoll, and British Shorthair were the top three most registered pedigree cat breeds, according to the Cat Fanciers Association. Similarly, in the United Kingdom, the British Shorthair, Ragdoll and Maine Coon were the most popular breeds registered in 2017 (71). Over the last 5 years, breeds with a decreased risk of obesity, such as the Oriental Shorthair, have proven less popular in those countries.

It therefore seems unlikely that the protective effect of purchasing a cat from a registered breeder is due to a predominance of breeds with decreased risk of obesity, in fact, the most popular registered breeds may be at an increased risk. It appears that this is a true effect, with further research required to determine exactly what role the breeder is playing and how they might be influencing the owner's behavior, feeding practices and choice of diet.

### Management-Related Risk Factors for Feline Obesity

Numerous dietary variables were significant risk factors for feline obesity. Two of these related to feeding a dry diet—feeding dry food purchased from a supermarket was a risk factor for obesity, and not feeding any dry food was protective. The latter finding is consistent with several (though not all) previous studies that have demonstrated that feeding a dry diet (as either all of the diet, or as the majority of the diet) is a risk factor for obesity in cats (44, 72). The increased risk associated with feeding a supermarket diet to cats has not been previously documented, in fact, studies by both Scarlett et al. (73) and Lund (8) revealed that overweight and obese cats were more likely to be fed a premium or therapeutic dry diet (8, 73). It was hypothesized that this finding may have been because of the increased fat content of premium therapeutic diets at that time.

These results are contrary to those of Suarez et al. who found that owners of overweight dogs were more interested in inexpensively priced commercial foods, and in discounted commercial foods or those with special offers (74). Interestingly, these owners also had less interest in the quality of the ingredients and nutritional composition of the commercial diets, when compared with owners of healthy weight dogs. Given that the fat content, energy density and palatability of most supermarket feline dry diets is now relatively comparable to most premium dry diets, it appears that this association between feeding a dry supermarket diet and increased risk of obesity might be an owner effect, rather than a diet effect. It may be the case that there are particular characteristics of owners that purchase dry food from supermarkets, such as a lack of time, motivation or disposable income, that increase the risk of feline obesity developing.

Specific types of raw feeding practices (but not all) were associated with a decreased risk of obesity in cats. Feeding human-grade raw meat (Model 3: OR 0.69, *p* = 0.002) or feeding raw bones (Model 3: OR 0.58, *p* = 0.022) was significantly protective, whereas feeding a raw commercial diet was not significant. Raw commercial diets are typically minced or ground meat-based products; owners that feed raw human-grade meat and raw bones may be more likely to be feeding chunks of different meat, bone and organs, although it is possible that they are also preparing a minced diet at home.

Regardless, given that feeding raw bones alone was strongly protective against obesity (Model 3), it may be the case that this type of diet provides beneficial enrichment, by allowing normal chewing and gnawing behaviors and extending the time taken for consumption of meals. Slower consumption of meals may decrease boredom, vocalizing and begging, and may also reduce stress and anxiety as well. It is certainly possible that the protective effect of feeding certain raw diets is therefore due to behavioral modification, rather than the composition of the diet itself. Alternatively, it may simply be that owners feeding a home-prepared raw meat-based diet are attentive to their cat's health and body condition. Preparation of a home-prepared raw diet can be time-consuming, expensive and inconvenient, and could indicate that owners prepared to undertake this are motivated, passionate about their cat's well-being, and highly focused on nutrition and weight management.

Feeding a home-prepared diet was also associated with a substantially decreased risk of obesity (Model 3: OR 0.49, *p* = 0.002); this may have been because owners of cats with a poor appetite and chronic disease are more likely to resort to a home-prepared diet to encourage increased food intake, when compared with owners of healthy cats. A limitation of this study is that no further information was requested on the nature of the home-prepared diet, so respondents may have selected this answer when feeding a cooked home-prepared diet for medical reasons, or alternatively, a standard, raw or cooked home-prepared diet.

Several common feline behaviors were shown to increase or decrease the risk of obesity; these included stealing human foods and stealing another cat's food. Hunting and eating prey was also significant using the visual scoring method (OR 0.71, *p* < 0.001) or the verbal scoring method (OR 0.78, *p* < 0.001), but not using cats that scored as obese using both methods. Cats that are hunting or stealing human foods may be fed less by their owner, or fed less palatable foods, decreasing the risk of excessive weight gain. Additionally, hunting occurs when cats are given outdoor access, which may be associated with increased activity and performance of natural feline behaviors, as previously discussed.

The inaccuracy when owners or veterinarians measure kibble with a scoop has been previously demonstrated (75, 76). Attempted measurement of small portion sizes results in the greatest degree of imprecision, with cats and small dogs at greater risk of overfeeding, when compared with larger animals (75). Many human studies also confirm that the size of food bowls, plates and utensils can impact the amount of food portioned and consumed (77, 78). It is therefore not surprising that using a scoop to measure out food was significantly associated with feline obesity in all three models (Model 3: OR 1.21, *p* = 0.019).

When BCS results using only the visual scoring method were used as the dependent variable, both weighing the amount of food dispensed, and adjusting the amount fed in response to changes in BCS were protective. This has not been previously demonstrated, however, weighing portion sizes is considered ideal for weight management (75). Regular assessment of BCS by owners and consequent adjustment of the amount fed also sounds ideal, however this requires education of owners so that they are, firstly, able to accurately and consistently detect subtle changes in body condition, and secondly, able to appropriately increase or decrease the amount fed per day, in response to any change. Many cat owners may lack the time or motivation to learn and implement this feeding approach; additionally, many veterinarians may also have insufficient time during consultations to provide this training.

### Discrepancy Between Analyses Using the Visual and Verbal Body Condition Scoring Methods

The three multivariable logistic regression models returned slightly different results. It is difficult to ascertain the reasons for these minor discrepancies. The additional results detected by the verbal scale may be partially due to the slightly higher number of respondents that scored their cat as either obese or overweight using the verbal scoring method, compared with the visual method. As previously mentioned, a limitation of this study is that the questionnaire did not ask for the cat's breed or coat length. Scoring body condition using a visual scale is inherently more subjective, when compared with a verbal scale. It is possible that owners of longhaired cats found the visual scale harder to use and that a greater degree of misclassification occurred with the visual scale.

Another contributing factor could be that humans rely on both verbal and the visual modes of thought, and there are individual preferences for verbal vs. visual thinking (79). It is possible that some participants may have responded more truthfully, and less emotionally, to the verbal BCS question, while some truly visual/spatial thinkers (believed to be <30% of the population) responded primarily (and with more accurate answers) to the visual BCS question (80).

It seems logical that, in this study, the most convincing results are, firstly, those that were found to be significant in all three models, and, secondly, the results of Model 3 alone, where any discordant BCS results had been removed from the analysis. This implies a lesser degree of confidence in the significance of six explanatory variables: owner preference for delayed reward (only Model 1), feeding supplements (only Model 2), weighing the amount fed (only Model 1), adjusting amount fed based on body weight (only Model 1), feeding a canned diet for health benefits (only Model 2) and feeding a canned diet that looks appealing to the owner (only Model 2). As mentioned previously, Colliard et al (4) found that a visual BCS scale was more accurate than a verbal BCS scale, with respect to owner-reported BCS. It is impossible to confirm or refute that, based on the results of this study, as both Model 1 and 2 correlate well with Model 3, producing relatively similar results.

## Conclusion

The results from this study emphasize the complexity of feline obesity, and the many contributing risk factors that may be associated with its development. There are clearly multiple cat-related, owner-related and management-related risk factors, and it is likely that a broad range of strategies and interventions will be required to substantially decrease the prevalence of this disease.

There are several key recommendations that arise from this research. Firstly, preventative efforts should be focused on the first year of the cat's life. Obesity typically develops early and is exacerbated by neutering; this emphasizes the essential role veterinarians may be able to play during this critical period, with respect to owner education and prevention of excessive weight gain. Secondly, encouraging owners to offer a range of different complete and balanced diets may be protective. Owners that enjoy feeding treats and find variety and indulgence appealing appear unlikely to be increasing the cat's risk of becoming overweight.

Thirdly, the guidance, education and practical advice provided by registered breeders may be of value to new cat owners. Breeders are well-positioned to support the owner during the cat's early life, and they should be encouraged to discuss obesity prevention with every client. Finally, all cat owners should be informed of the benefits of environment enrichment for indoor cats; this is likely to provide a range of behavioral and health benefits, one of which may be a decreased risk of obesity.

It is well-recognized that obesity in people is such a complex problem that no single intervention is likely to make a significant difference. Recommendations include countering the obesogenic environment and focusing on critical stages throughout life, with coordinated, multisectoral action. Similarly, it is essential to recognize the central role of the owner, and for veterinarians, breeders and owners to work together with the goal of reducing the risk of this serious, yet completely preventable, disease in cats.

## Data Availability

The datasets generated for this study are available on request to the corresponding author.

## Author Contributions

MW contributed to the design, to the conduction of the study, to the analysis and interpretation of the results, and to the writing of the manuscript. NC contributed to the design, interpretation of the results, and review of the manuscript. EV contributed to the design, statistical analysis, interpretation of the results, and review of the manuscript.

### Conflict of Interest Statement

The authors declare that the research was conducted in the absence of any commercial or financial relationships that could be construed as a potential conflict of interest.

## Footnotes

^1^Images taken from the Global Nutrition Committee Toolkit, provided courtesy of the World Small Animal Veterinary Association: https://www.wsava.org/WSAVA/media/PDF_old/Body-condition-score-chart-cats.pdf
