# Supplementary material for: Owner and Cat-Related Risk Factors for Feline Overweight or Obesity
Source: Front Vet Sci. 2019 Aug 19;6:266. doi: 10.3389/fvets.2019.00266 (PMC6709657; doi:10.3389/fvets.2019.00266)
Supplement: Supplementary file 1 [file Table_1.DOCX]

**Supplementary material A.** Questions for an online questionnaire administered to cat owners in various social media groups, from March to May 2017.

**Part 1: Questions on cat health**

**1) What is your cat’s name?**

**2) What country do you come from?**

**3) How old is your cat?**

Less than 1 year old

1-4 years

5-8 years

9-12 years

13-16 years

Older than 16 years

**4) What is your cat’s gender?**

Male (desexed)

Male (not desexed)

Female (desexed)

Female (not desexed)

**5) Does your cat live?**

Indoors only

Mainly indoors with restricted outdoor access e.g. courtyard, on leash

Indoors and free to roam outdoors

Outdoors with restricted indoor access

Outdoors only

**6) Where did you get your cat from?**

A registered breeder (member of a breed association, national stud register etc.)

An unregistered breeder

A pet store

A friend or family member

A rescue group, shelter or center

My cat was a stray

The pound

A private seller that advertised online

**7) Which picture does your cat look most like? Pick the best option.**Please consider carefully and be honest; this is an anonymous questionnaire and there are no wrong answers.


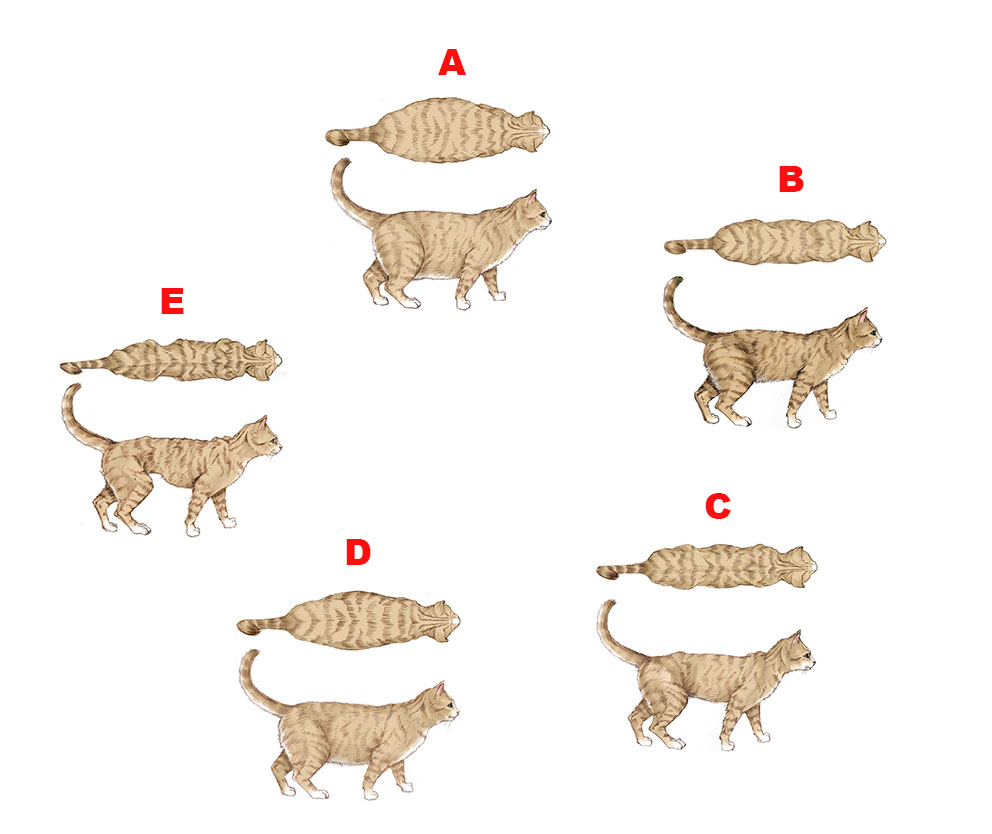


*[Question on next page and not seen by owner until after answering visual scale question above]*

**How would you describe your cat? Pick the best option.**Please consider carefully and be honest; this is an anonymous survey and there are no wrong answers**.**

Very underweight *(ribs can be seen and felt, hip bones and spine easily seen and felt)*

Slightly underweight *(ribs can be felt very easily, hip bones and spine felt easily)*

A healthy weight *(ribs felt with light pressure, waist visible from above and from the side)*

Slightly overweight *(ribs not easily felt, not much waist, belly a bit rounded or saggy between back legs)*

Very overweight *(ribs can’t be felt, no waist, belly rounded and saggy)*

Obese *(ribs can’t be felt, lots of fat over hips and chest, no waist, rounded large belly)*

**Part 2: Big Five Inventory (10 items)**

**How well do the following statements describe your personality?**

| I see myself as someone who … | SD | D | N | A | SA |
| --- | --- | --- | --- | --- | --- |
|  |  |  |  |  |  |
| Is reserved |  |  |  |  |  |
| Is generally trusting |  |  |  |  |  |
| Tends to be lazy |  |  |  |  |  |
| Is relaxed, handles stress well |  |  |  |  |  |
| Has few artistic interests |  |  |  |  |  |
| Is outgoing, sociable |  |  |  |  |  |
| Tends to find fault with others |  |  |  |  |  |
| Does a thorough job |  |  |  |  |  |
| Gets nervous easily |  |  |  |  |  |
| Has an active imagination |  |  |  |  |  |

SA = Strongly Agree

A = Agree

N = Neutral

D = Disagree

SD = Strongly Disagree

**Part 3: Lexington Attachment to Pets Scale**

**Please tell us whether you agree or disagree with some very brief statements about your cat.**

|  | SD | D | N | A | SA |
| --- | --- | --- | --- | --- | --- |
| My cat means more to me than any of my friends |  |  |  |  |  |
| Quite often I confide in my cat |  |  |  |  |  |
| I believe that cats should have the same rights and privileges as family members |  |  |  |  |  |
| I believe my cat is my best friend |  |  |  |  |  |
| Quite often, my feelings towards people are affected by how they react to my cat |  |  |  |  |  |
| I love my cat because he/she is more loyal to me than most of the people in my life |  |  |  |  |  |
| I enjoy showing other people pictures of my cat |  |  |  |  |  |
| I think my cat is just a cat |  |  |  |  |  |
| I love my cat because it never judges me |  |  |  |  |  |
| My cat knows when I’m feeling bad |  |  |  |  |  |
| I often talk to other people about my cat |  |  |  |  |  |
| My cat understands me |  |  |  |  |  |
| I believe that loving my cat helps me stay healthy |  |  |  |  |  |
| Cats deserve as much respect as humans do |  |  |  |  |  |
| My cat and I have a very close relationship |  |  |  |  |  |
| I would do almost anything to take care of my cat |  |  |  |  |  |
| I play with my cat quite often |  |  |  |  |  |
| I consider my cat to be a great companion |  |  |  |  |  |
| My cat makes me feel happy |  |  |  |  |  |
| I feel that my cat is a part of my family |  |  |  |  |  |
| I am not very attached to my cat |  |  |  |  |  |
| Owning a cat adds to my happiness |  |  |  |  |  |
| I consider my cat to be a friend |  |  |  |  |  |

SA = Strongly Agree

A = Agree

N = Neutral

D = Disagree

SD = Strongly Disagree

**Part 4: Brief self-control scale (13-items)**

**How well do the following statements describe you?**

|  | SD | D | N | A | SA |
| --- | --- | --- | --- | --- | --- |
|  |  |  |  |  |  |
| I have a hard time breaking bad habits |  |  |  |  |  |
| I get distracted easily |  |  |  |  |  |
| I say inappropriate things |  |  |  |  |  |
| I do certain things that are bad for me, if  they are fun |  |  |  |  |  |
| I’m good at resisting temptation |  |  |  |  |  |
| People would say that I have very strong  self-discipline |  |  |  |  |  |
| Pleasure and fun sometimes keep me from  getting work done |  |  |  |  |  |
| I do things that feel good in the moment but  regret later on |  |  |  |  |  |
| I am able to work effectively toward long-term goals |  |  |  |  |  |
| Sometimes I can’t stop myself from doing  something, even if I know it is wrong |  |  |  |  |  |
| I refuse things that are bad for me |  |  |  |  |  |
| I often act without thinking through all the  alternatives |  |  |  |  |  |
| I am lazy |  |  |  |  |  |
| I wish I had more self-discipline |  |  |  |  |  |

SA = Strongly Agree

A = Agree

N = Neutral

D = Disagree

SD = Strongly Disagree

**Part 5: Consideration of Future Consequences-14 Scale**

**How well do the following statements describe you?**

|  | SD | D | N | A | SA |
| --- | --- | --- | --- | --- | --- |
|  |  |  |  |  |  |
| I consider how things might be in the future, and try to influence those things with my day to day behavior |  |  |  |  |  |
| Often I engage in a particular behavior in order to achieve outcomes that may not result for many years |  |  |  |  |  |
| I only act to satisfy immediate concerns, figuring the future will take care of itself |  |  |  |  |  |
| My behavior is only influenced by the immediate (i.e., a matter of days or weeks) outcomes of my actions |  |  |  |  |  |
| My convenience is a big factor in the decisions I make or the actions I take |  |  |  |  |  |
| I am willing to sacrifice my immediate happiness or well-being in order to achieve future outcomes |  |  |  |  |  |
| I think it is important to take warnings about negative outcomes seriously even if the negative outcome will not occur for many years |  |  |  |  |  |
| I think it is more important to perform a behavior with important distant consequences than a behavior with less important immediate consequences |  |  |  |  |  |
| I generally ignore warnings about possible future problems because I think the problems will be resolved before they reach crisis level |  |  |  |  |  |
| I think that sacrificing now is usually unnecessary since future outcomes can be dealt with at a later time |  |  |  |  |  |
| I only act to satisfy immediate concerns, figuring that I will take care of future problems that may occur at a later date |  |  |  |  |  |
| Since my day to day work has specific outcomes, it is more important to me than behavior that has distant outcomes |  |  |  |  |  |
| When I make a decision, I think about how it might affect me in the future |  |  |  |  |  |
| My behavior is generally influenced by future consequences |  |  |  |  |  |

SA = Strongly Agree

A = Agree

N = Neutral

D = Disagree

SD = Strongly Disagree

**Part 6: Indulgent Feeding Style Questionnaire**

**How true are these statements for you?**

| Item | SD | D | N | A | SA |
| --- | --- | --- | --- | --- | --- |
| I feed my cat when it seems unhappy |  |  |  |  |  |
| I reward my cat with food when it does something I like |  |  |  |  |  |
| I get my cat something else to eat straight away if it doesn’t like its food |  |  |  |  |  |
| My cat deserves to have treats |  |  |  |  |  |
| My cat decides when it wants to eat |  |  |  |  |  |
| I like to offer my cat new foods to try |  |  |  |  |  |
| If my cat won’t eat it’s food I usually try something different after a few days |  |  |  |  |  |
| I buy a certain cat food because I think my cat would find it tasty |  |  |  |  |  |
| My cat decides how much it eats |  |  |  |  |  |
| I make sure my cat always has food available |  |  |  |  |  |
| I praise my cat for eating all its food |  |  |  |  |  |
| My cat lets me know when it is hungry |  |  |  |  |  |
| I decide when my cat eats |  |  |  |  |  |
| My cat eats whatever I offer it |  |  |  |  |  |

SA = Strongly Agree

A = Agree

N = Neutral

D = Disagree

SD = Strongly Disagree

**Part 7: Cat Feeding Practices**

**1) How often do you feed your cat?**

One meal a day

Two meals a day

Three meals a day

My cat always has access to dry food

My cat always has access to wet food

**2) What do you currently feed your cat? (*Check all that apply*)**

Dry food (from supermarket)

Dry food (from pet store)

Dry food (from my vet)

Canned food (from supermarket)

Canned food (from pet store)

Canned food (from my vet)

Veterinary diet for a medical condition

Grain-free dry food

Organic commercial pet foods

Weight loss diet (either dry or canned)

Raw meat diet (commercial pet)

Raw meat diet (human-grade and home-prepared)

Raw bones

Human-grade fresh or frozen seafood

Freeze-dried or rehydrated commercial pet foods

Home-prepared pet food

Commercial pet treats

Human food treats

Table scraps

Supplements

Plants

Other

**3) I feed my cat dry food because … (*check all that apply*)**

I don’t feed my cat dry food

My cat prefers it to all other foods

I think my cat likes crunchy foods

It is a cheaper option

I like the packaging

It has health benefits for my cat

I think my cat likes the shape and size of the biscuits

It’s convenient and easy to feed

I think the flavors sound appealing

It doesn’t make any mess

It doesn’t smell

My cat’s breeder recommended dry food

My vet recommended dry food

Other

**4) I feed my cat canned food because … (*check all that apply*)**

I don’t feed my cat canned food

It’s my cat’s favorite type of food

It has health benefits for my cat

I like the ingredients

I think the food looks like it would be tasty to a cat

I like the packaging

I think my cat likes soft foods

It is a cheaper option

It’s convenient and easy to feed

I like the sound of the flavors

My cat’s breeder recommended canned food

My vet recommended canned food

Other

**5) I can’t really control what my cat eats**

True

False

**6) If true: I can’t control what my cat eats because … (*check all that apply*)**

My cat hunts and eats what it catches

My cat eats the neighbor’s cat’s food

Other family members feed my cat

Friends or flat mates feed my cat

My cat steals human food

My cat steals my other cat’s food

**7) How much do you vary your daily feeding routine? (*Check all that apply*)**

My cat gets the same food everyday

My cat often gets different types of canned food but the same dry food

My cat mainly gets a range of different canned foods

My cat often gets different raw meats or bones

My cat gets different dry foods quite frequently (every 1-2 weeks)

I feed my cat different foods all the time e.g. meat, canned and dry cat food, scraps, or treats

**8) How often do you give your cat treats?**

Everyday

A few times a week

Once a week

Very rarely

Never

**9) How true are these statements for you?**

|  | SD | D | N | A | SA |
| --- | --- | --- | --- | --- | --- |
| I typically feed my cat around the same time every day |  |  |  |  |  |
| I typically feed my cat the same amount every day |  |  |  |  |  |
| I typically feed my cat the same type of food every day |  |  |  |  |  |

SA = Strongly Agree

A = Agree

N = Neutral

D = Disagree

SD = Strongly Disagree

**10) How do you work out how much to feed your cat? (Check all that apply)**

I follow the recommendation on the packaging

I follow the recommendation of my cat's breeder

I follow my vet's recommendation

I use a measuring cup to ensure my cat gets the right amount

I weigh my cat's food to ensure he/she gets the right amount

My cat gets a certain number of cans per day

My cat always has food available

I change the amount I feed my cat depending on whether he/she is gaining or losing weight

**Part 8: Owner socio-demographic characteristics**

**1) What is your gender?**

Male

Female

Prefer not to say

**2) What is your age?**

20 years and under

21-30 years

31-40 years

41-50 years

51-60 years

61 years and over

Prefer not to say

**3) What level of education have you completed?**

Primary school (less than 7 years)

Junior high school (7-10 years)

High school (university entrance qualification)

Vocational training or apprenticeship

University (Bachelor degree)

University (Post-graduate degree)

Prefer not to say

**4) Which of the following statements about occupational status apply to you?**

Not working at the moment

Looking for work

Part-time or hourly work (< 15 hours per week)

Part-time work (15 to 34 hours per week)

Full-time work

On temporary leave (education leave, public service leave)

In training (apprentice)

Studying full time (university or private college)

Prefer not to say

**5) I currently live in**

A large city

A large town

A small town or village

In the country

Prefer not to say
