# Supplementary material for: Owner and Cat-Related Risk Factors for Feline Overweight or Obesity
Source: Front Vet Sci. 2019 Aug 19;6:266. doi: 10.3389/fvets.2019.00266 (PMC6709657; doi:10.3389/fvets.2019.00266)
Supplement: Supplementary file 2 [file Table_2.DOCX]

**Supplementary material B.** Name and description of variables analysed as risk factors for owner-reported feline overweight/obesity.

| **Variable name** | **Variable levels** |
| --- | --- |
| CAT CHARACTERISTICS | |
| Cat’s age | < 1yr  1-4 years  5-8 years  9-12 years  13-16 years  > 16 years |
| Cat’s gender | Male entire (ME)  Female entire (FE)  Male neutered (MN)  Female neutered (FN) |
| Housing | Outdoors only  Indoors only  Indoors/restricted outdoors  Indoors/outdoors  Outdoors/restricted indoors |
| Source | Registered breeder  Unregistered breeder  Pet store  Friend/family  Rescue group/shelter  Stray  Pound  Private online seller |
| OWNER VARIABLES | |
| Owner gender | Male  Female  Prefer not to say |
| Owner age | < 20 years  21-30 years  31-40 years  41-50 years  51-60 years  > 60 years Prefer not to say |
| Owner level of education | Primary school  Junior high school  High school  Vocational training  University (Bachelor)  University (Post-graduate)  Prefer not to say |
| Owner employment status | Not working at the moment Looking for work Part-time < 15hrs/week Part-time 15-35hrs/week Full-time Temporary leave Training (apprenticeship) Studying full-time Prefer not to say |
| Owner location | Rural  Small town  Large town  Large city  Prefer not to say |
| Owner conscientiousness | High  Low |
| Owner neuroticism | High  Low |
| Owner openness | High  Low |
| Owner agreeableness | High  Low |
| Attachment to cat | Low  Medium High Very High |
| Owner self-control | Low  Medium High Very High |
| Owner preference for immediate reward | High  Low |
| Owner preference for delayed reward | High  Low |
| Management Variables | |
| Owner’s use of indulgent feeding practices | High Low |
| Owner’s use of consistent feeding practices | High  Low |
| Treat feeding | Never  Rarely  Sometimes  Often  Always |
| Same food fed everyday | Y/N |
| Different canned/same dry fed | Y/N |
| Range of canned foods fed | Y/N |
| Different raw meats/bones fed | Y/N |
| Different dry fed all the time | Y/N |
| Very wide range of foods fed | Y/N |
| Cat hunts and eats prey | Y/N |
| Steals from neighbors | Y/N |
| Fed by family | Y/N |
| Fed by friends/flat mate | Y/N |
| Steals human food | Y/N |
| Steals other cat’s food | Y/N |
| Dry food diet – supermarket | Y/N |
| Dry food diet – pet store | Y/N |
| Dry food diet – vet | Y/N |
| Dry food diet – grain free | Y/N |
| Wet food diet – supermarket | Y/N |
| Wet food diet – pet store | Y/N |
| Wet food diet – vet | Y/N |
| Veterinary/prescription diet | Y/N |
| Organic commercial diet | Y/N |
| Weight loss (wet or dry) | Y/N |
| Raw meat (commercial raw pet diet) | Y/N |
| Raw meat (human grade and home-prepared) | Y/N |
| Raw bones | Y/N |
| Freeze-dried/rehydrated | Y/N |
| Seafood (human grade) | Y/N |
| Home-prepared diet | Y/N |
| Commercial treats | Y/N |
| Human food treats | Y/N |
| Table scraps | Y/N |
| Supplements | Y/N |
| Plants | Y/N |
| Don’t feed dry food at all | Y/N |
| Feed dry food because: cat prefers it | Y/N |
| Feed dry food because: cat likes crunchy food | Y/N |
| Feed dry food because: cheaper | Y/N |
| Feed dry food because: owner likes the packaging | Y/N |
| Feed dry food because: perceived health benefits | Y/N |
| Feed dry food because: convenient | Y/N |
| Feed dry food because: flavors sound appealing to owner | Y/N |
| Feed dry food because: no mess | Y/N |
| Feed dry food because: breeder recommended it | Y/N |
| Feed dry food because: vet recommended it | Y/N |
| Don’t feed canned food at all | Y/N |
| Feed canned food because: cat prefers it | Y/N |
| Feed canned food because: cheaper | Y/N |
| Feed canned food because: owner likes the packaging | Y/N |
| Feed canned food because: owner likes ingredients | Y/N |
| Feed canned food because: food looks tasty to owner | Y/N |
| Feed canned food because: perceived health benefits | Y/N |
| Feed canned food because: convenient | Y/N |
| Feed canned food because: flavors sound appealing to owner | Y/N |
| Feed canned food because: soft texture | Y/N |
| Feed canned food because: breeder recommended it | Y/N |
| Feed canned food because: vet recommended it | Y/N |
| Feeding method: Package recommendation | Y/N |
| Feeding method: Breeder’s recommendation | Y/N |
| Feeding method: Vet’s recommendation | Y/N |
| Feeding method: Measuring cup | Y/N |
| Feeding method: Weigh on scales | Y/N |
| Feeding method: Certain number of cans | Y/N |
| Feeding method: Ad lib feeding | Y/N |
| Feeding method: Adjusted for body weight | Y/N |
