# Supplementary material for: Owner and Cat-Related Risk Factors for Feline Overweight or Obesity
Source: Front Vet Sci. 2019 Aug 19;6:266. doi: 10.3389/fvets.2019.00266 (PMC6709657; doi:10.3389/fvets.2019.00266)
Supplement: Supplementary file 5 [file Table_5.DOCX]

**Supplementary material E.** Univariable logistic regression model results for risk factors for feline overweight/obesity (results from all cats scored as overweight using both the visual and verbal scoring methods [n = 1607] compared with all cats scored as not overweight using both methods [n=4134]).

| Variable name | Category | Coefficient | SE | P value | OR (CI) | LRT |
| --- | --- | --- | --- | --- | --- | --- |
| CAT VARIABLES | |  |  |  |  |  |
| Age | < 1yr  1-4 years  5-8 years  9-12 years  13-16 years  > 16 years | -1.6969 1.1095  1.7173 1.7069  1.3316 0.7301 | 0.0957 0.1023  0.1058  0.1118 0.1227  0.1619 | < 2e-16  < 2e-16  < 2e-16  < 2e-16  6.49e-06 | Ref.  3.03 (2.49–3.72)  5.56 (4.54–6.87)  5.51 (4.43–6.88)  3.78 (2.98–4.82)  2.07 (1.51–2.84) | < 2.2e-16 |
|  |  |  |  |  |  |  |
| Gender | ME  FE  MN  FN | -1.3926 -0.0310  1.0463 0.9834 | 0.1789 0.2303  0.1817 0.1819 | 0.893 8.53e-09  6.50e-08 | Ref.  0.96 (0.61–1.53)  2.84 (2.01–4.11)  2.67 (1.89–3.86) | < 2.2e-16 |
| Housing | Outdoors only  Indoors only  Indoors/restricted outdoors  Indoors/outdoors  Outdoors/restricted indoors | -0.6286 0.2061  0.1706  0.2349 -0.0935 | 0.4378 0.4389  0.4404  0.4400 0.5282 | 0.639 0.699  0.593 0.859 | Ref.  1.23 (0.53–3.05)  1.18 (0.51–2.95)  1.26 (0.54–3.15)  0.91 (0.32–2.64) | 0.6639 |
| Source | Registered breeder  Unregistered breeder  Pet store  Friend/family  Rescue group/shelter  Stray  Pound  Private online seller | -1.1915 0.5725  1.0614  0.9407 0.9021  0.8858  1.1740 0.6407 | 0.0626 0.1683  0.1460  0.0874 0.0752  0.0868  0.1125 0.1168 | 0.000673  3.58e-13  < 2e-16  < 2e-16  < 2e-16  < 2e-16  4.18e-08 | Ref.  1.77 (1.26–2.45)  2.89 (2.16–3.84)  2.56 (2.15–3.04)  2.46 (2.12–2.85)  2.42 (2.04–2.87)  3.23 (2.59–4.03)  1.89 (1.50–2.28) | < 2.2e-16 |
| OWNER VARIABLES | |  |  |  |  |  |
| Owner gender | Male  Female  Prefer not to say | -1.0644 -0.0725 -0.6583 | 0.1021 0.1062 0.3581 | 0.494 0.066 | Ref. 0.93 (0.75–1.14) 0.51 (0.24–1.00) | 0.1082 |
| Owner age | < 20 years  21-30 years  31-40 years  41-50 years  51-60 years  > 60 years Prefer not to say | -1.0824 -0.2061 0.0507 -0.0351 -0.0066 -0.0049 -0.3038 | 0.1270 0.1389 0.1404 0.1405 0.1445 0.1584 0.3756 | 0.138 0.718 0.802 0.963 0.975 0.419 | Ref. 0.81 (0.62–1.07) 1.05 (0.80-–1.39) 0.96 (0.74–1.27) 0.99 (0.73–1.32) 0.99 (0.73–1.36) 0.73 (0.33–1.48) | 0.3871 |
| Owner education | Primary school  Junior high school  High school  Vocational training  University (Bachelor)  University (Post-graduate)  Prefer not to say | -1.1134 0.3679 0.3122 0.3478 0.2123 0.3021  0.3234 | 1.1579 1.1623 1.1598 1.1588 1.1536 1.1512  1.1667 | 0.771 0.827 0.709 0.874 0.803  0.811 | Ref. 1.38 (0.19–31.6) 1.29 (0.15–26.8) 1.35 (0.21–29.4) 1.21 (0.16–26.0) 1.36 (0.19–27.5)  1.42 (0.18–27.5) | 0.4890 |
| Owner employment | Not working at the moment  Looking for work  Part-time < 15hrs/week  Part-time 15-35hrs/week  Full-time  Temporary leave  Training (apprenticeship)  Studying full-time  Prefer not to say | -0.7003  -0.0589 -0.1226 -0.1671  -0.0899 0.1212 -1.1378  -0.1996 0.0178 | 0.0708  0.1804 0.1522 0.1027  0.0838 0.2779 0.6119  0.1211 0.1365 | 0.778 0.534 0.166  0.302 0.698 0.069  0.076 0.927 | Ref.  0.91 (0.59–1.26) 0.84 (0.64–1.18) 0.82 (0.71–1.03)  0.89 (0.79–1.02) 1.14 (0.70–1.89) 0.34 (0.06–0.93)  0.88 (0.61–1.07) 0.96 (0.79–1.26) | 0.1812 |
| Owner location | Rural  Small town  Large town  Large city  Prefer not to say | -0.7855 0.0273 0.1032 0.0019 0.0064 | 0.0916 0.1100 0.1145 0.0966 0.2844 | 0.803 0.376 0.951 0.967 | Ref. 1.10 (0.81–1.19) 1.16 (0.92–1.26) 0.97 (0.89–1.18) 1.04 (0.59–1.75) | 0.3744 |
| Owner conscientiousness | High  Low | -0.4799  0.1528 | 0.0326  0.0522 | 0.00346 | Ref. 1.16 (1.05–1.29) |  |
| Owner neuroticism | Low  High | -0.4652 0.0418 | 0.0359 0.0503 | 0.406 | Ref.  1.04 (0.94–1.15) |  |
| Owner agreeableness | Low  High | -0.4865 0.0689 | 0.0512 0.0592 | 0.244 | Ref. 1.07 (0.95–1.20) |  |
| Owner openness | Low  High | -0.4499 0.0397 | 0.0368 0.0503 | 0.43 | Ref. 1.04 (0.94–1.14) |  |
| Owner attachment | Low  Medium High Very High | -0.3659 -0.0309 -0.1126 -0.0388 | 0.1984 0.2016 0.2019 0.2066 | 0.878 0.576 0.850 | Ref.  0.96 (0.65–1.44) 0.89 (0.60–1.33) 0.87 (0.64–1.09) | 0.3908 |
| Owner self-control | Low  Medium High Very High | -0.3462 -0.0316 -0.1103 -0.3366 | 0.2426 0.2450 0.2448 0.3366 | 0.897 0.652 0.556 | Ref. 0.96 (0.60–1.57) 0.89 (0.55–1.45) 0.82 (0.42–1.58) | 0.3751 |
| Owner preference for immediate reward | Low  High | -1.1538 -0.0497 | 0.0397 0.0585 | 0.395 | Ref. 1.05 (0.94–1.18) |  |
| Owner preference for delayed reward | Low  High | -1.0981 -0.0408 | 0.0455 0.0596 | 0.493 | Ref.  0.96 (0.85–1.07) |  |
| MANAGEMENT VARIABLES | |  |  |  |  |  |
| Indulgent feeding | Low  Medium High Very high | -0.6818 -0.0947 -0.3616 -0.5178 | 0.1254 0.1323 0.1312 0.2112 | 0.48983 0.00769 0.01421 | Ref. 0.90 (0.69–1.19) 0.69 (0.53–0.91) 0.59 (0.39–0.88) | 5.6e-06 |
| Consistent feeding | High  Low | -1.0330  -0.2629 | 0.0421 0.0727 | 0.000303 | Ref. 0.76 (0.66–0.88) | 0.0003031 |
| Treat feeding | Never  Very rarely  Once a week  A few times a week  Everyday | -0.9644 -0.1656 -0.0584 -0.2799 -0.1637 | 0.0972 0.1105 0.1246 0.1117 0.1159 | 0.1340 0.6389 0.0123 0.1577 | Ref. 0.84 (0.68–1.05) 0.94 (0.73–1.20) 0.75 (0.59–0.94) 0.84 (0.67–1.06) | 0.001067 |
| Feeding routine | Same food everyday  Different canned/same dry  Range of canned  Different raw meats/bones  Different dry  Wide range of foods | 0.3644 0.0408  -0.2797 -0.8473  0.0318 -0.0380 | 0.054 0.058  0.099 0.115  0.110 0.100 | 1.77e-11 0.487  0.00486 1.94e-13  0.774 0.705 | 1.43 (1.29–1.60) 1.04 (0.92–1.17)  0.75 (0.62–0.91) 0.42 (0.34–0.53)  1.03 (0.82–1.27) 0.96 (0.78–1.17) |  |
| Control over feeding | Cat hunts  Steals from neighbors  Fed by family  Fed by friends/flat mate  Steals human food  Steals other cat’s food | -0.0756  0.3919  0.1610  0.0302  -0.2358  0.4187 | 0.074  0.131 0.118  0.243  0.103  0.092 | 0.311  0.00282 0.175  0.901  0.0222  6.24e-06 | 0.92 (0.79–1.07)  1.48 (1.14–1.90) 1.17 (0.92–1.47)  1.03 (0.62–1.63)  0.78 (0.64–0.96)  1.52 (1.26–1.82) |  |
| Diet | Dry food – supermarket  Dry food – pet store  Dry food – vet  Dry food – grain free  Wet food – supermarket  Wet food – pet store  Wet food – vet  Veterinary diet  Organic commercial  Weight loss (wet or dry)  Commercial raw  Raw meat (human grade)  Raw bones  Seafood (human grade)  Freeze-dried/rehydrated  Home-prepared  Commercial treats  Human food treats  Table scraps  Supplements  Plants | 0.3220  0.0498  0.1654 -0.1530  0.0905 -0.2208 -0.0496  0.2286 -0.2946  2.2874  -0.4347 -0.7578  -1.1981 -0.2808  -0.8213  -1.0051 0.1193  -0.0127  0.0311  -0.5423  -0.0789 | 0.058  0.057  0.074  0.072 0.058  0.064  0.120  0.101  0.157 0.161  0.106 0.091  0.183 0.130  0.182  0.200  0.060  0.095  0.097  0.124  0.143 | 4.52e-08  0.385  0.0267 0.0336  0.122 0.000662 0.679  0.024 0.0606  <2e-16  4.19e-05 <2e-16  6.85e-11 0.032 6.9e-06 5.41e-07 0.05 0.894  0.751  1.26e-05 0.584 | 1.37 (1.23–1.54)  1.05 (0.93–1.17)  1.17 (1.02–1.36)  0.85 (0.74–0.98)  1.09 (0.97–1.22)  0.80 (0.70–0.91)  0.95 (0.74–1.20)  1.25 (1.02–1.52)  0.74 (0.54–1.00)  9.84 (7.24–13.63)  0.64 (0.52–0.79)  0.46 (0.39–0.55)  0.30 (0.20–0.42)  0.75 (0.58–0.97)  0.43 (0.30–0.62)  0.36 (0.24–0.53)  1.12 (0.99–1.26)  0.98 (0.81–1.18)  1.03 (0.84–1.24)  0.58 (0.45–0.73)  0.92 (0.69–1.22) |  |
| Dry food feeding | Don’t feed it at all  Cat prefers it  Cat likes crunchy food  Cheaper  Like the packaging  Health benefits  Convenient  Flavors sound appealing  No mess  Breeder recommended it  Vet recommended it | -0.7810  0.1951  0.1841  0.3840  -0.7141  0.0784  0.1841  0.1778  0.1869  -1.0495  0.0812 | 0.119  0.075  0.073  0.086  0.546  0.055  0.055  0.122  0.091  0.177  0.065 | 5.98e-11 0.00991  0.0121  8.86e-06  0.191 0.157  0.000922 0.145 0.0417 3.46e-09  0.215 | 0.45 (0.36–0.57)  1.22 (1.05–1.41)  1.20 (1.04–1.38)  1.46 (1.24–1.73)  0.48 (0.14–1.28)  1.08 (0.96–1.20)  1.20 (1.08–1.34)  1.19 (0.93–1.51)  1.21 (1.01–1.44)  0.35 (0.24–0.48)  1.08 (0.95–1.23) |  |
| Canned food feeding | Don’t feed it at all  Cat prefers it  Cheaper Like the packaging  Like the ingredients  Food looks tasty  Soft texture  Health benefits  Convenient  Flavors sound appealing  Breeder recommended it  Vet recommended it | 0.2155  -0.0358  -0.0965  0.3291  -0.1629  0.1040  0.0302  -0.1340  -0.1340  0.0018  -1.5278  -0.0105 | 0.059  0.063  0.238  0.332  0.094  0.078  0.069  0.061  0.061  0.129  0.279  0.083 | 0.000319 0.575 0.686 0.322 0.0852  0.183  0.661  0.0281 0.814  0.989  4.69e-08  0.899 | 1.24 (1.10–1.39)  0.96 (0.85–1.09)  0.90 (0.55–1.42)  1.38 (0.70–2.62)  0.84 (0.70–1.02)  1.10 (0.95–1.29)  1.03 (0.89–1.18)  0.87 (0.77–0.98)  0.97 (0.82–1.16)  1.00 (0.77–1.28) 0.21 (0.11–0.36)  0.98 (0.83–1.16) |  |
| Feeding method/amount | Package recommendation  Breeder’s recommendation  Vet’s recommendation  Measuring cup  Weigh on scales  Certain number of cans  Ad lib feeding  Adjusted for body weight | -0.0251  -0.9644  0.2407  0.4036  -0.6309  -0.0059  -0.0696  -0.1712 | 0.067  0.230  0.065  0.061  0.142  0.074  0.056  0.070 | 0.708  2.81e-05  0.000242  6.41e-11  9.46e-06  0.937  0.217  0.0145 | 0.97 (0.85–1.11)  0.38 (0.23–0.58)  1.27 (1.11–1.44)  1.50 (1.33–1.69)  0.53 (0.39–0.69)  0.99 (0.85–1.15)  0.93 (0.83–1.04)  0.84 (0.73–0.96) |  |
